# Familial *DHCR7* genotype presenting as a very mild form of Smith‐Lemli‐Opitz syndrome and lethal holoprosencephaly

**DOI:** 10.1002/jmd2.12155

**Published:** 2020-08-09

**Authors:** Suzanna E. L. Temple, Rani Sachdev, Carolyn Ellaway

**Affiliations:** ^1^ Centre for Clinical Genetics Sydney Children's Hospital Randwick New South Wales Australia

**Keywords:** 7‐dehydrocholesterol, *DHCR7*, holoprosencephaly, Smith‐Lemli‐Opitz syndrome

## Abstract

Smith‐Lemli‐Opitz syndrome (SLOS) is an autosomal recessive metabolic disorder caused by variants in the *DHCR7* gene. In cholesterol biosynthesis, 7‐dehydrocholesterol (7‐DHC) is converted to cholesterol by the enzyme 7‐DHC reductase, which is encoded by the gene *DHCR7*. Thus, an elevated 7‐DHC is indicative of SLOS. Characteristically SLOS is usually associated with congenital anomalies, dysmorphisms, and moderate to severe neurodevelopmental delay. However, there are rare descriptions of individuals with milder phenotypes. We report a mild case of SLOS presenting with short stature, cleft palate, imperforate anus, and mild language delay with subtle dysmorphic features. 7‐DHC was not elevated at 1 year of age and SLOS considered excluded at this time. The parents had two pregnancies with holoprosencephaly. Whole exome sequencing of one of the fetuses identified compound heterozygous pathogenic variants in the *DHCR7* gene (c.964‐1G>C (p.?) and c.1039G>A (p.Gly347Ser) causative of SLOS. The proband with a mild form of SLOS was also found to have the same *DHCR7* variants as the fetus and repeat testing of 7‐DHC at 4 years of age was elevated, in keeping with SLOS. This case is the first to describe a wide intrafamilial phenotypic spectrum of SLOS as a result of the same *DHCR7* genotype. This case also supports the findings of others that a normal or near normal development should not exclude SLOS. As demonstrated in this case exclusion of a metabolic diagnosis because of a negative biochemical marker such as 7‐DHC is not absolute and if clinical suspicion remains genomic sequencing is warranted.

**SYNOPSIS**

Factors other than *DHCR7* genotype influence an individual's Smith‐Lemli‐Opitz syndrome (SLOS) phenotype as evidenced by a familial *DHCR7* genotype presenting as mild neurodevelopmental disability and fatal holoprosencephaly. Furthermore, exclusion of SLOS based on normal 7‐dehydrocholesterol, as occurred in the mild form of SLOS, is not conclusive, and if clinical suspicion remains genomic sequencing is recommended.

## 1 INTRODUCTION

Smith‐Lemli‐Opitz syndrome (SLOS; OMIM 270400) is a congenital multiple anomaly syndrome caused by an abnormality in cholesterol metabolism; pathogenic variants in the *DHCR7* gene (OMIM 602858)^1^ result in a deficiency of the enzyme, 7‐dehydrocholesterol (7‐DHC) reductase. The prevalence of SLOS is estimated to be approximately 1:20000 to 1:60000^1^ with an overall carrier frequency of 1.4%, highest in Ashkenazi Jews (1 in 43) and Northern Europeans (1 in 54).^2^ Comparing predicted birth incidence with that observed in the literature suggests that 42% to 88% of affected conceptuses experience prenatal demise.^2^

SLOS has a broad phenotypic spectrum characteristically associated with prenatal and postnatal growth retardation, microcephaly, moderate to severe intellectual disability, and congenital anomalies. Observed features include characteristic facial features, cleft palate, abnormal gingivae, cardiac defects, hypospadias, ambiguous genitalia, postaxial polydactyly and 2–3 toe syndactyly. Individuals with rarer, milder forms may have only subtle facial characteristics, such as hypotonia, 2–3 toe syndactyly, and mild to no intellectual disability.^1^

The diagnosis of SLOS relies on clinical suspicion and early detection of elevated 7‐DHC. The decreased activity of the enzyme 7‐DHC reductase, results in the inability to convert 7‐DHC to cholesterol resulting in increased 7‐DHC.^3^ While most individuals also have hypocholesterolemia it is not always useful for diagnosis as approximately 10% of affected individuals have normal cholesterol levels particularly when individuals are older or have a milder phenotype.^4^ Most studies have shown an inverse correlation between serum concentration of cholesterol and clinical severity with mortality particularly high in the group with the lowest cholesterol concentrations.^5^, ^6^

*DHCR7* is the only gene in which pathogenic variants are known to cause SLOS and approximately 96% of known pathogenic variants are detected on sequence analysis of *DHCR7* gene.^1^ A strict genotype‐phenotype correlation is difficult because most individuals are compound heterozygotes.^1^ Waterham and Hennekam,^5^ following review of 207 individuals with SLOS, found that those with the most severe phenotypes had two null variants or two variants in loops 8 to 9.

We report a case demonstrating the wide phenotypic spectrum of SLOS (mild phenotype to antenatal holoprosencephaly) within the same family carrying the same familial pathogenic *DHCR7* variants. In addition the mild form of SLOS was not identified on early biochemical testing of serum 7‐DHC and only found to be elevated following genetic diagnosis of SLOS.

## 2 CASE PRESENTATION

The proband, 5‐year‐old boy, was born to healthy nonconsanguineous Caucasian parents. He was naturally conceived and born via normal vaginal delivery at 37 weeks gestation following induction of labor, after steroid cover, for maternal cholestasis. Antenatal ultrasounds at 12, 20, and 35 weeks reported normal growth and development. His birth weight was 2.78 kg (3rd‐15th centile), length 46.5 cm (3rd centile) and occipitofrontal circumference 33 cm (3rd‐15th centile). Apgar scores were 9 at 1 minute and 10 at 5 minutes.

He was noted at birth to have a submucous cleft palate and required a cleft palate teat for feeding until surgical repair at 9 months of age. He had tympanometry and bilateral grommet insertion concurrent with cleft palate repair for left mild conductive hearing impairment thought to be due to cleft palate. He required repeat grommet insertion at 2 years old for chronic otitis media.

Following discharge after birth, he was readmitted to hospital with irritability and diagnosed with imperforate anus, which was surgically repaired on day 15 of life. This initially required regular manual dilatation and was subsequently replaced with stool softener for management of constipation. Investigations for possible VATER association, including spinal X‐ray, and skeletal survey were normal. An echocardiogram reported a patent foramen ovale, which subsequently resolved. A renal ultrasound demonstrated mild pelvicalyceal dilatation with a normal maturating cystourethrogram.

Postnatally, he demonstrated symmetrical growth restriction with weight, height, and head circumference below or on the second centile. He had early feeding issues including gastro‐oesophageal reflux that was treated with changes in formula, medication, and at times nasogastric tube feeds. Endoscopy showed mild gastro‐oesophageal reflux disorder. At 5 years of age, his weight was 13.3 kg (0.46 percentile; *Z* score −2.61), height was 96.2 cm (first percentile; *Z* score −2.29), and head circumference was 47 cm (0.93 percentile; *Z* score 2.35).

While developmentally appropriate in all other areas, he was noted to have expressive language delay from 12 months of age. At 2 years and 9 months, he had only learnt to say one to two words but was able to follow complex instructions. He continues to receive speech therapy and attends a mainstream school with support. At 4 years of age, he developed sleeping issues that improved with melatonin. There were no significant behavioral issues and he was a gentle, social boy with no signs of hyperactivity.

**FIGURE 1:**
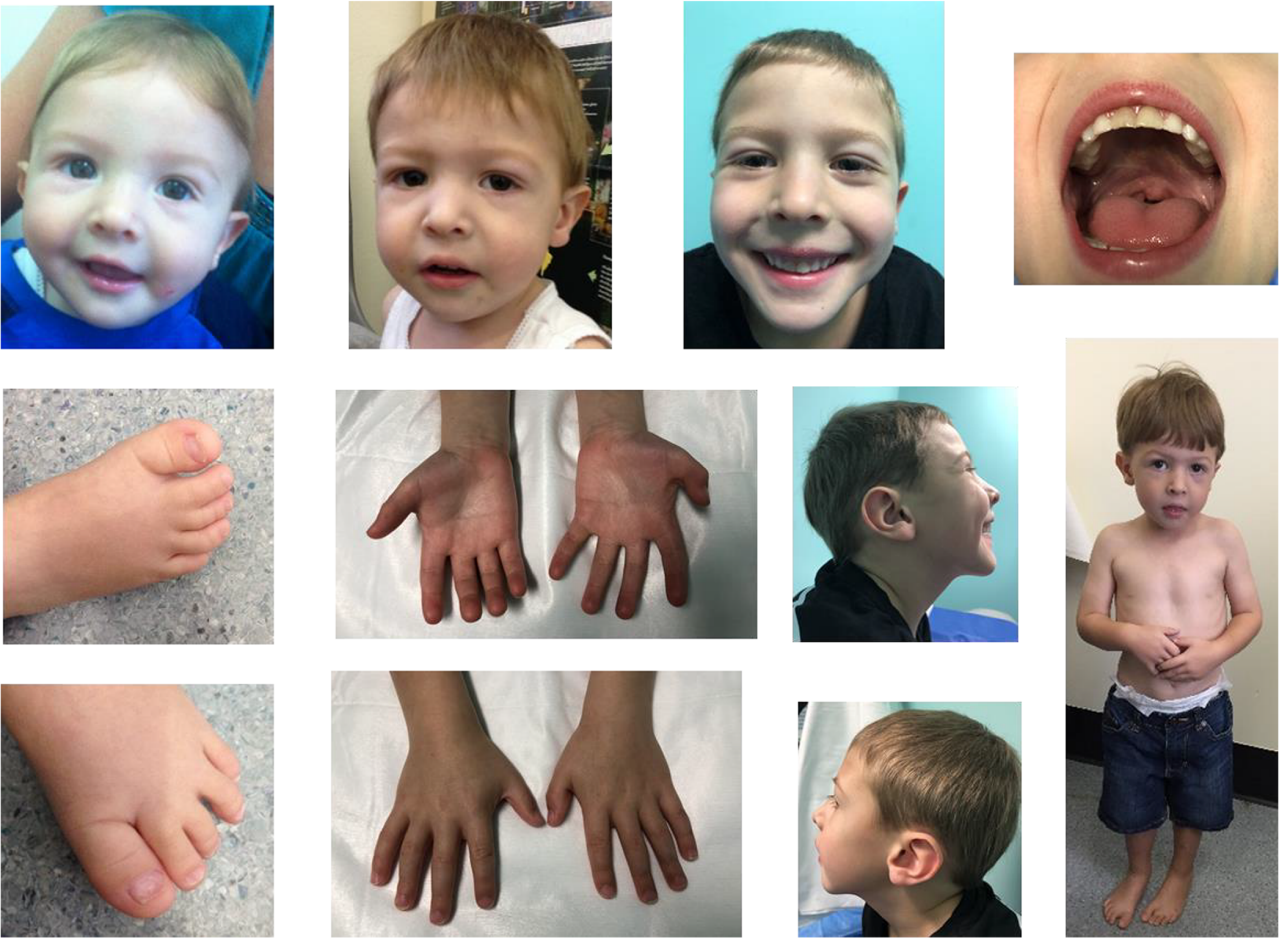
The features of the proband, at 18‐24 months old, include down‐slanting palpebral fissures, slightly low‐set and prominent ears, bilateral transverse creases, pectus excavatum and bilateral two to three toes syndactyly

On examination, he was noted to have down‐slanting palpebral fissures, broad nasal bridge, slightly low‐set and prominent ears, bilateral single transverse palmar creases, pectus excavatum, and bilateral 2–3 toe syndactyly (Figure 1). The remainder of the examination was unremarkable.

The biochemical marker for SLOS, 7‐DHC, was tested at 12 months of age to exclude SLOS based on the combination of microcephaly, mild language delay, cleft palate, and 2–3 toes syndactyly. Levels of 7‐DHC and cholesterol were reported as within normal range. A chromosomal array was normal. Thyroid function, growth hormone, cortisol, and adrenocorticotropic hormone were all reported as within normal range.

**FIGURE 2:**
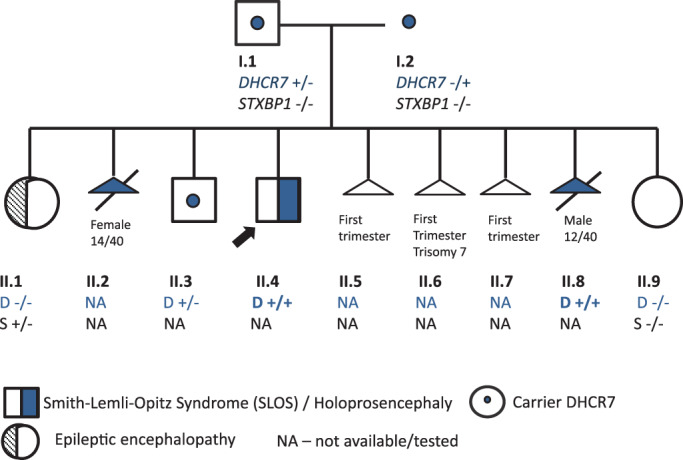
Pedigree. Proband highlighted with arrow (II.4). Three first trimester miscarriages (11.5‐11.7); one with trisomy 7 on karyotype (II.6). Two pregnancies were ceased for holoprosencephaly on antenatal ultrasound; female at 14 weeks gestation (II.2) and male at 12 weeks gestation (II.8). Older sister had severe epileptic encephalopathy due to a de novo heterozygous *STXBP1* variant (II.1) not carried by any other family member (S −/−). Smith‐Lemli‐Opitz syndrome (SLOS) associated compound heterozygous *DHCR7* variants present in proband (arrow) and II.8 (D+/+). Carrier of *DHCR7* (D+/−)

The proband's family history was complex with his parents having nine pregnancies (Figure 2), including three first trimester miscarriages (Figure 2, II.5‐II.7) with one found to have trisomy 7 on karyotype (Figure 2, II.6). The karyotypes of both parents were normal. Two pregnancies were terminated for holoprosencephaly demonstrated on antenatal ultrasounds—a female with a normal karyotype on amniocentesis (Figure 2, II.2) and a male with a normal chromosomal array on chorionic villus sampling (Figure 2, II.8). The boy had three siblings; an older healthy brother (Figure 2, II.3), a younger sister with left multicystic kidney (Figure 2, II.9), and an older sister with severe epileptic encephalopathy due to a de novo heterozygous *STXBP1* variant (Figure 2, II.1).

Trio whole exome sequencing of the parents and the fetus of the eighth pregnancy (Figure 2, II.8) was performed to identify the cause of the recurrent holoprosencephaly. Clinical trio whole exome sequencing including deletions and duplications was performed at Fulgent Diagnostics (Temple City, California). A phenotype‐driven analysis of the submitted specimen was undertaken, focusing on genes associated with holoprosencephaly, neurodevelopmental delay, seizures. The results of this study identified biparentally inherited compound heterozygous pathogenic variants in the *DHCR7* gene (paternally inherited *DHCR7* NM_001360.2 c.964‐1G>C (p?), rs138659167, and maternally inherited *DHCR7* NM_001360.2 c.1039G>A (p.Gly347Ser), HGMD: CM051456, causative of autosomal recessive SLOS and an explanation for holoprosencephaly in the two pregnancies (Figure 2, II.2 and II.8).

While 7‐DHC testing had not previously been suggestive of SLOS the constellation of the features and the detection of the familial *DHCR7* variants necessitated genetic testing. The same familial compound heterozygous variants in *DHCR7* were also identified in the proband following targeted variant testing at Fulgent Genetics (DNA was amplified for the target region and sequenced bidirectionally). Repeat 7‐DHC testing at 4 years of age was elevated, 36 μmol/L (normally undetected) consistent with SLOS while plasma cholesterol was normal at 4.5 mmol/L (3.0‐5.5 mmol/L). The same laboratory (Biochemical Genetics, Westmead Children's Hospital) performed cholesterol and 7‐DHC testing on the earlier and repeat sample using the same methodology (GC‐MS system). From 2015, the laboratory was also able to determine the presence of 8‐DHC (unable to quantitate), and 8‐DHC was detected in the repeat sample.

The younger brother was found to be a carrier of the paternally inherited *DHCR* 7 c.964‐1G>C variant. The older sister, with severe epileptic encephalopathy (Figure 2, II.1), did not carry either of the familial *DHCR7* variants and had a normal MRI brain.

The proband started treatment with a cholesterol supplement, at 2000 mg per day (140 mg/kg/d) to help improve growth and development.^7^ Repeat 7‐DHC levels 16 months after commencement of cholesterol supplementation were significantly reduced (12 μmol/L).

## 3 DISCUSSION

*DHCR7*, located on chromosome 11, contains nine exons with exons 3 to 9 encoding the protein 7‐dehydrocholesterol reductase.^8^ The protein consists of nine putative trans‐membrane helices and a sterol‐sensing domain. More than 150 pathogenic variants have been described with the majority (84%) missense variants distributed among all coding exons.^5^ As with this family, the majority of SLOS patients (approximately 60%) have a combination of one missense and one nonsense variant and approximately 30% have two missense variants.^5^

The paternally inherited *DHCR7* variant, c.964‐1G>C (p?), is one of the most common variants in Caucasians (allele frequency of approximately 30%), and disrupts the canonical splicing acceptor site adjacent to the last exon 9^9^ predicted to result in a non‐functional protein, and is associated with a severe phenotype.^5^ The maternally inherited *DHCR7* variant, c.1039G>A p.Gly347Ser, is a missense variant located in exon 9 encoding the transmembrane 8 region of the protein. This variant has previously been reported in trans with a common pathogenic nonsense variant (p.Trp151*) in a male patient diagnosed at 7 months old with moderately severe SLOS.^10^ A combination of these familial nonsense and missense *DHCR7* variants, both located in loops 8 to 9, would be predicted to be associated with the more severe SLOS phenotype^5^ but not holoprosencephaly or mild SLOS.

Only approximately 5% of individuals with SLOS have holoprosencephaly^11^, ^12^ and it represents the most severe form of this syndrome. No specific *DHCR7* genotype‐phenotype correlation has been identified but most reported cases carry two null variants.^11^, ^12^, ^13^, ^14^ However, there have been other reported cases of homozygous null variants in *DHCR7* with no reported holoprosencephaly.^14^ Weaver et al^14^ speculated that concomitant variants in *DHCR7* and one of the genes causing nonsyndromic holoprosencephaly, including *SHH* (sonic hedgehog gene) may be the underlying mechanism resulting in holoprosencephaly. This is unlikely to be the mechanism in this case as reanalysis was recently performed on the whole exome sequencing of the parents and fetus of the eighth pregnancy and did not identify any variants in genes associated with holoprosencephaly including *CDON*, *GLI2*, *PTCH1*, *SHH*, *SIX3*, *TGIF1*, and *ZIC2*. Environmental and maternal factors were also suggested to influence resulting phenotype.^14^

The proband is one of a very small cohort of children with SLOS who have normal to mild developmental delay. More than 25 cases of mild SLOS in the literature have been reported with none having the same missense variant as in our proband.^15^, ^16^, ^17^ Two adult brothers with mild SLOS carried the same nonsense variant as our proband with a missense variant also located in loops 8 to 9.^17^ They both had elevated 7‐DHC and normal cholesterol and presented with microcephaly, short stature, bilateral 2–3 toe syndactyly, language delay, intellectual disability, and late aggressive behavior with one brother having a cleft palate.^17^ Their parents had two pregnancies, which resulted in still births.^17^ All reported mild cases had nonfamilial 2–3 toe syndactyly with more than half having subtle facial appearance (ptosis, short‐upturned nose, microcephaly), feeding issues, poor weight gain, developmental delay, and behavioral issues.^15^, ^16^, ^17^, ^18^ All cases had an elevated 7‐DHC although sometimes only slightly increased and all had normal cholesterol.^15^, ^16^, ^17^ In several families stillbirths or miscarriages occurred,^15^, ^16^, ^17^ which raises the question as to whether other families with a familial *DHCR7* genotype also have family members presenting at the extremes of the SLOS phenotype. Many of the mild cases of SLOS were suspected and diagnosed only following recognition of the condition in a family member.^15^ The phenotypic presentation of the mild form of SLOS may be so minimal that the diagnosis is not considered or excluded based on a normal 7‐DHC as in this case. This raises the possibility that the real incidence of SLOS may be higher than described not only with regard to the severe lethal form but also for the mild form.

Individuals, from different families, with the same *DHCR7* genotype can have markedly different phenotypes in terms of severity and plasma 7‐DHC levels.^5^ However, this is the first reported case of this documented within a family. Many have suggested that SLOS is a Mendelian disorder which appears to be significantly influenced by other genetic, epigenetic, and environmental factors.^15^, ^19^, ^20^ The maternal‐fetal cholesterol transfer during pregnancy is dependent on maternal diet, maternal apolipoprotein levels, and probably several other factors, and has been postulated to have a significant impact on the SLOS phenotype.^21^ Others suggest that although SLOS is caused only by mutations in the *DHCR7* gene, the SLOS phenotype appears to be greatly influenced by variants in many as yet unidentified genes.^22^

We report a familial pathogenic *DHCR7* genotype presenting at the extreme ends of the SLOS phenotypic spectrum. In the same family, one affected child had mild dysmorphic features and language delay while two other pregnancies were affected with holoprosencephaly and all likely a result of the same *DHCR7* genotype. This bimodal distribution of clinical severity associated with the same familial genotype suggests that other factors, in addition to genotype, significantly influence an individual's phenotype including maternal factors, fetal synthesis of cholesterol by an alternative metabolic pathway, altered expression of the alleles or modifier genes, or epigenetics. Unfortunately, there are no biochemical results for the two holoprosencephaly pregnancies as this may have been helpful in determining the cause of the differing effects of the same familial genotype. The diagnosis of SLOS in the proband further highlights the difficulty in diagnosing mild forms of SLOS and the likelihood that others have been undetected and the important role that genetic sequencing has over biochemical testing in cases of clinically suspected SLOS.

## CONFLICT OF INTEREST

The authors declare no potential conflict of interest.

## COMPLIANCE WITH ETHICS GUIDELINES

Written informed consent was obtained from the patient's parents for publication of this case report and any accompanying images. Manuscript was written by first author, S .E. L.T. All authors have contributed to manuscript production, and reviewed the material, and agreed to its content. Ethics approval and funding were not required for this study.
